# Early life exposure to cigarette smoking and adult and old-age male mortality: Evidence from linked US full-count census and mortality data

**DOI:** 10.4054/demres.2023.49.25

**Published:** 2023-10-11

**Authors:** Jonas Helgertz, John Robert Warren

**Affiliations:** 1 Lund University School of Economics and Management, Lund, Sweden.; 2 University of Minnesota Twin Cities, Minneapolis, MN, USA.

## Abstract

**BACKGROUND:**

Smoking is a leading cause of premature death across contemporary developed nations, but few longitudinal individual-level studies have examined the long-term health consequences of exposure to smoking.

**OBJECTIVE:**

We examine the effect of fetal and infant exposure to exogenous variation in smoking, brought about by state-level cigarette taxation, on adulthood and old-age mortality (ages 55–73) among cohorts of boys born in the United States during the 1920s and 1930s.

**METHODS:**

We use state-of-the-art methods of record linkage to match 1930 and 1940 US full-count census records to death records, identifying early life exposure to the implementation of state-level cigarette taxes through contemporary sources. We examine a population of 2.4 million boys, estimating age at death by means of OLS regression, with post-stratification weights to account for linking selectivity.

**RESULTS:**

Fetal or infant exposure to the implementation of state cigarette taxation delayed mortality by about two months. Analyses further indicate heterogenous effects that are consistent with theoretical expectations; the largest benefits are enjoyed by individuals with parents who would have been affected most by the tax implementation.

**CONCLUSIONS:**

Despite living in an era of continuously increasing cigarette consumption, cohorts exposed to a reduction in cigarette smoking during early life enjoyed a later age at death. While it is not possible to comprehensively assess the treatment effect on the treated, the magnitude of the effect should not be underestimated, as it is larger than the difference between having parents belonging to the highest and lowest socioeconomic groups.

**CONTRIBUTION:**

The study provides the first estimates of long-run health effects from early life exposure to cigarette smoking.

## 1. Introduction

Smoking is a leading cause of premature death across contemporary developed nations. The physiological pathways linking the inhalation of cigarette smoke to short- and long-term health effects have been well established by observational studies of humans (Doherty et al. 2009; Peto et al. 2000; Pietinalho, Pelkonen, and Rytilä 2009; Postma, Bush, and van den Berge 2015) and by animal experiments (Hecht 2005; Vlahos and Bozinovski 2018). Research has also suggested intergenerational effects, with maternal first- or secondhand smoking linked to adverse outcomes among infants and children, in part through negative effects on the fetal development process. Fetuses of smoking mothers experience a significantly higher risk of being born preterm or small for gestational age (Jaddoe et al. 2008), and exposure to cigarette smoking while in utero is linked to obesity, high blood pressure, and a range of respiratory outcomes in childhood (Banderali et al. 2015; Jaakkola and Gissler 2004; Russell, Taylor, and Maddison 1966; Von Kries et al. 2002). Illustrative of the rapid physiological development that occurs during the immediate postnatal phase, exposure to secondhand smoking during this period has been linked to infant death (Anderson and Cook 1997) and also to impaired neurodevelopment (Herrmann, King, and Weitzman 2008) and respiratory health (Håberg et al. 2007).

An important weakness characterizing the existing body of research is a lack of studies demonstrating long-term (i.e., decades-long) consequences of early life exposure to cigarette smoking. We contribute to the literature by estimating the association between early life exposure to the implementation of state-level cigarette taxation from the 1920s to the 1940s in the United States and adult and old-age mortality. Our study uses state cigarette taxes to proxy for short-term reductions in maternal first- or secondhand cigarette smoking, and our results indicate that fetal and infant exposure to a short-term reduction in cigarette smoking is associated with a moderate increase in life expectancy – amounting to about two months. Our results also indicate heterogeneity in these associations across population subgroups, with more substantial life expectancy gains among groups expected to have experienced the largest reductions in exposure. Our results are not only of scientific relevance but are also of substantial policy importance, as exposure to cigarette smoking remains an important source of fetal exposure to a range of hazardous substances (Drake, Driscoll, and Mathews 2018; Treyster and Gitterman 2011).

## 2. Literature review

The health consequences of using tobacco products are well-known, with the Centers for Disease Control and Prevention reporting that nearly one in five deaths in the United States is due to cigarette smoking (Centers for Disease Control and Prevention 2021). Empirical research over decades, primarily focusing on firsthand smoking, has shown this to substantially increase the risk of several types of cancers (Gaudet et al. 2013; Nomura et al. 2012; Peto et al. 2000) and other diseases that either shorten life expectancy or at the very least have a substantial negative influence on quality of life, such as chronic obstructive pulmonary disease (Postma et al. 2015) and asthma (Pietinalho, Pelkonen, and Rytilä 2009). Health fears concerning the use of cigarettes and tobacco more broadly have existed since long before they were scientifically proven, with warnings issued by medical professionals and other prominent figures as early as the 1800s. However, it was not until the 1960s that sufficient medical evidence had accumulated to allow the US surgeon general to conclusively state that smoking represents a health hazard through being causally linked to lung cancer (Brandt 2007).^3^

Early studies examined aggregated statistics, finding a rather robust link between tobacco prices, consumption, or taxes and various health outcomes. One of the earliest studies displayed a strong correlation between cigarette sales at the state level and lung cancer mortality rates, both observed in 1950 (Friedman 1967). This finding was complemented by Schoenberg and colleagues’ (1971) study of the association between state-level cigarette and alcohol consumption and mortality due to esophageal cancer between 1950 and 1966; they found a moderate correlation (0.64) between per capita cigarette sales and esophageal cancer mortality rates. Breslow and Enstrom (1974) used similar data and found that cigarette consumption is the factor most closely related to respiratory cancers. Since then, studies have been able to exploit individual-level data as well as more sophisticated methods of analysis, typically examining how individuals’ own use of cigarettes is correlated with their concurrent and future health outcomes. Most research demonstrates associations between smoking and a plethora of adverse health outcomes, from poor lung function (Anthonisen, Connett, and Murray 2002) to increased risk of death due to diseases ranging from ischemic heart disease to various cancers (Bjartveit and Tverdal 2005).

Studies linking fetal or infant exposure to cigarette smoking ‒ through the mother’s first- or secondhand smoking – have to our knowledge never studied long-term outcomes. Banderali et al. (2015) nevertheless outline that existing research has been able to link fetal exposure to parental smoking to several adverse postnatal health outcomes. These include preterm birth, fetal growth restriction, low birth weight, sudden infant death syndrome, neurodevelopmental and behavioral problems, obesity, hypertension, type 2 diabetes, impaired lung function, asthma, and wheezing. Other correlational studies, using Scandinavian administrative register data, have found increased risk of premature birth (Kyrklund-Blomberg and Cnattingius 1998) as well as asthma (Jaakkola and Gissler 2004). A smaller literature has explicitly focused on the consequences of exposure during the first years of life; findings also support the existence of a range of independent negative health effects. Looking separately at the effects of pre- and postnatal exposure to secondhand smoking on a range of child conduct outcomes, several studies have found independent effects from the latter (Glenn, Ragno, and Liu 2023). Studies have also found links between exposure to secondhand smoking during infancy and lung function (Vanker, Gie, and Zar 2017).

Lien and Evans 2005 is one of few studies that approach the subject from a causal perspective. The authors exploit cigarette tax hikes across four US states as a vehicle for assessing the influence of maternal smoking on the birth weight of exposed babies. While finding an effect on birth weight and on the probability of being born low birth weight, they caution against over-interpreting the magnitude of the effects. Similar findings regarding infant mortality were obtained by Sen and Piérard (2011) using time-series data on Canadian provinces between 1979 and 2004. In a study by Evans and Ringel (1999), the authors demonstrate how increased excise taxes on cigarettes result in both reduced smoking participation among pregnant women and an increase in the average birth weight of children. Lastly, Simon (2016) examines fetal exposure to state cigarette tax hikes during the period 1989–2009, focusing on childhood health as the outcome. The findings show that third-trimester exposure to an increase in taxes results in both fewer school sick days and a lower probability of having to visit the doctor twice or more during a 12-month period.

## 3. Theoretical framework: How early life exposure to maternal smoking might affect children’s longevity

The link between fetal and infant exposure to cigarette smoke and adverse long-term health outcomes is best understood through the fundamental role these periods play in an individual’s physiological development. Maternal smoking during or shortly after pregnancy may substantially disrupt the developmental trajectory of the fetus or child and is also associated with a range of adverse birth outcomes. Not only does it increase the risk of preterm birth (Ion and Bernal 2015; Shah and Bracken 2000; Wisborg et al. 1996), but it is also the main preventable cause of low birth weight: full-term infants of smoking mothers weigh on average 200 grams (about 7 ounces) less that children of nonsmoking mothers (Abel 1980; Jaddoe et al. 2008; Kramer 1987). Some research into the mechanisms through which fetal and infant exposure to maternal smoking causes postnatal health adversity emphasizes its irreversible consequences for lung development. And while an individual’s lung development continues well into adolescence (Kotecha 2000; Schittny 2017), the key phase for the structural development and functioning of the lungs is confined to the fetal stage and the first two years of life (Gibbs, Collaco, and McGrath-Morrow 2016). Children of mothers who smoked during pregnancy^4^ manifest worse pulmonary function according to a range of different parameters (Gilliland et al. 2000), with many effects persisting through childhood (Kalliola et al. 2013) and into adulthood (Harding and Maritz 2012). This supports the hypothesis that disruption to lung development has permanent scarring effects. In addition, maternal smoking is associated with an increased risk of a range of children’s respiratory illnesses and asthma (Kalliola et al. 2013; Yarnell and St Leger 1979; Zacharasiewicz 2016). Finally, animal experiments have shown lung structure to deteriorate with age more rapidly among subjects exposed to cigarette smoke while in utero (Harding and Maritz 2012).

Although at least 4,000 harmful substances are delivered through cigarette smoke (Knopik et al. 2012), the transmission of nicotine (Bruin, Gerstein, and Holloway 2010; Rehan, Asotra, and Torday 2009) and carbon monoxide (Venditti, Casselman, and Smith 2011) emerges as particularly important in understanding the mechanisms through which fetal exposure to cigarette smoking may permanently affect fetuses’ lung development. Fetal exposure to nicotine occurs through its ability to cross the placental barrier and to contaminate the amnionic fluid; the fetus thus ingests nicotine and absorbs it through the skin. By acting as a vasoconstrictor, nicotine adversely influences supplies of nutrients and oxygen (Lambers and Clark 1996). This then interferes with several aspects of lung development, including decreasing overall lung volume, which causes the development of fewer but larger air saccules and airway hyper-responsiveness, resulting in airflow restriction. In addition, longer-run consequences suggest emphysematous changes in the more rapidly aging lung. Fetuses of mothers who smoke also display higher levels of carbon monoxide in their blood, which prevents hemoglobin from transporting oxygen, increases the risk of fetal hypoxia, and disrupts enzymes engaged in intracellular respiration, possibly resulting in disturbed fetal growth.

### 3.1 Cigarette smoking in the United States

We use the state-level implementation of cigarette taxes in the United States in the first half of the 20^th^ century to investigate the association between early life exposure to cigarette smoke and long-term mortality outcomes. The fundamental premise of our strategy is that new state-level taxes on cigarettes temporarily reduce maternal consumption – and thus temporarily reduce fetal and infant exposure.

At the time of the invention of the mass-produced cigarette in the 1890s, tobacco had already been consumed in America for centuries. While never without opponents, on either moral or health grounds, other ways of ingesting tobacco – as chew, as snuff, or in a pipe or cigar – remained dominant a few decades into the 20^th^ century. Through advances in the mass manufacturing of cigarettes, powerful lobbying, and effective, aggressive, and targeted advertising especially toward the young and women (Brandt 2007), cigarette smoking had become the dominant type of tobacco consumption in the United States by the end of the 1930s (Milmore and Conover 1956).

From the 1910s and until the 1960s, the average number of cigarettes smoked per individual in the United States increased at an almost uninterrupted pace. Widespread commercial advertising depicted cigarette smoking as associated with generally desirable characteristics, such as sociability and attractiveness. With the lack of knowledge about smoking’s negative health consequences (Brandt 2007), the average American’s consumption of cigarettes increased from less than 5 packages per year to almost 150 packages per year (Orzechowski and Walker 2014).^5^ The growing popularity of the cigarette during the first two decades of the 20^th^ century was noteworthy given the initial difficulties facing the product on the US market; no fewer than 15 states entirely prohibited cigarette sales at some point between 1896 and 1921. However, the enforcement of such laws was lax in the face of massive public demand. The most employed method of restricting the use of cigarettes was to prohibit their sale to minors – a step that had been implemented by every state except Texas by 1940 (Alston, Dupré, and Nonnenmacher 2002).

We begin our analysis in 1920, the beginning of the golden age of the cigarette in America – a time when cigarette use increased dramatically and permeated virtually all segments of the population. Much of the overall increase in rates of tobacco use in the early 20^th^ century was due to the increasing popularity of cigarettes – particularly among women (Brandt 2007). Despite large increases in rates of cigarette smoking among women, men remained more frequent cigarette smokers through the first half of the 20^th^ century, consistent with the earlier stages of the cigarette epidemic model of Lopez, Collishaw, and Piha (1994). Consequently, during the period in question, secondhand smoking was likely to have been the most common source of exposure to cigarette smoke for married women.

According to a supplement to the 1955 Current Population Survey, age-adjusted proportions of current adult cigarette smokers amounted to about 60% of men and 27% of women (Haenzel, Shimkin, and Miller 1956). The same data suggest that socioeconomic differences in smoking prevalence at the time were quite different from what they are today, with negligible differences overall between socioeconomic groups. Indeed, the share of male regular cigarette smokers in the high-status occupation group “managers, officials, and proprietors” was essentially the same as in the low-status occupation groups “laborers” and “unemployed” (ibid: 62). A more evident difference emerges between groups depending on whether they were engaged in agriculture-related activities. Whereas 59% of farmers and farm managers and 62% of farm laborers and foremen, respectively, were regular smokers (ibid: 64), the share among individuals engaged outside farming was distinctly higher, hovering at around 70%.^6^ This is also reflected in geographical patterns, with smoking largely being an urban phenomenon (Friedman 1967). However, this is confounded by rural environments being characterized by farming to a larger extent. In fact, Haenszel and colleagues (1956: 58) report negligible differences between urban and rural areas that are not characterized by farming activities, with 69% and 68% of men, respectively, being regular smokers. For women, a similar picture emerges, with 30% and 27% of urban and rural nonfarm residents, respectively, being regular smokers.

### 3.2 Cigarette taxation in the United States

**Figure 1:**
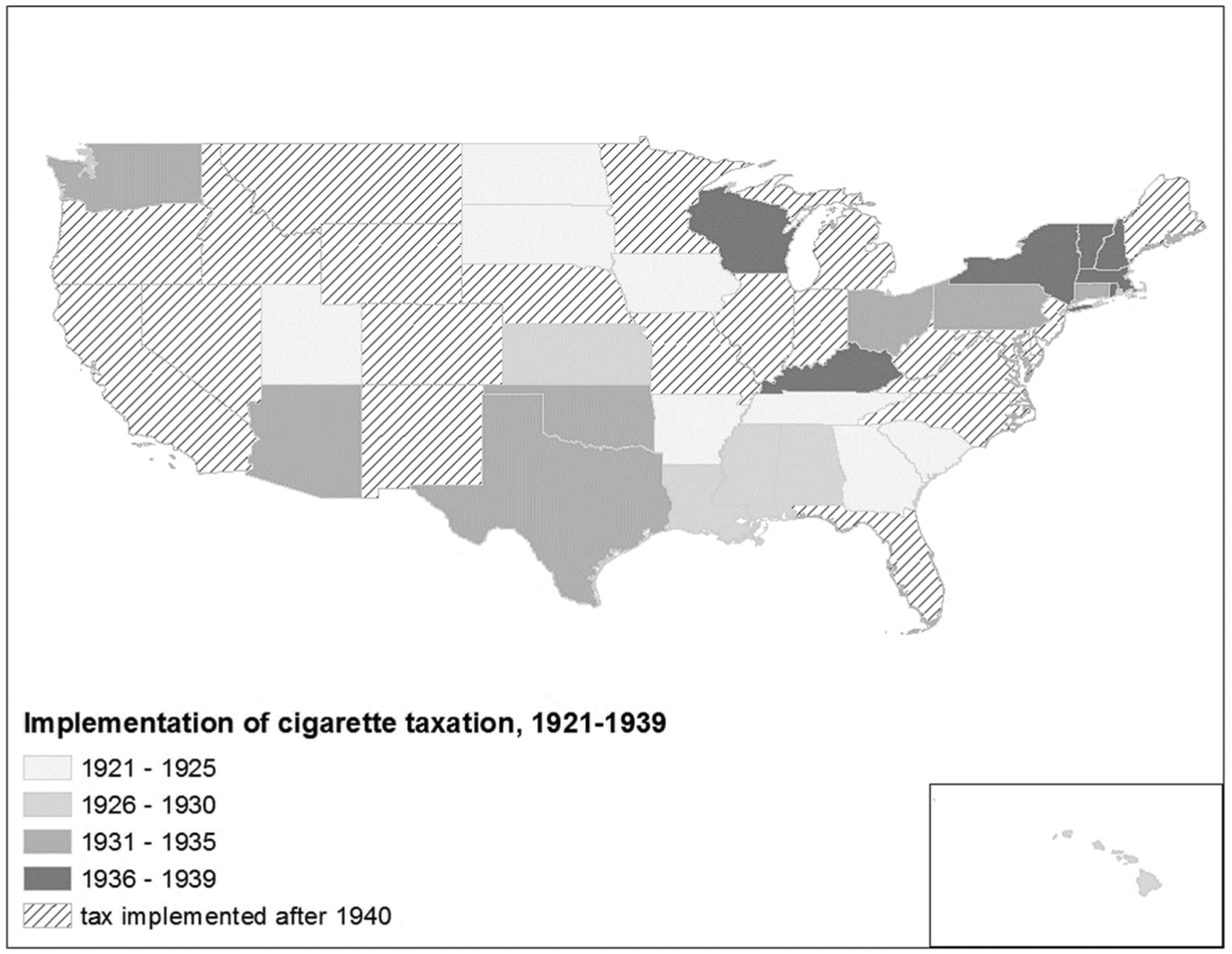
Timing of state-level cigarette taxation *Source*: Orzechowski and Walker 2014.

Taxes on tobacco in the United States were initially introduced on fiscal grounds, with federal taxation preceding taxes levied at the state and city levels. The federal government’s first national tobacco tax was introduced to raise funds during the Civil War (Advisory Commission on Intergovernmental Relations “State-Federal Overlapping in Cigarette Taxes” 1964); however, it remained in effect after the war’s end. Consequently, by the time Iowa implemented the first state-level cigarette tax in 1921, it was excised on top of an existing federal tax of $3 per 1,000 cigarettes (or 6¢ per pack of 20). During the period we examine, the federal cigarette tax remained constant (Orzechowski and Walker 2014), so the principal source of exogenously induced change in the price of cigarettes across states was through the introduction of state cigarette taxes.^7^ Iowa’s pioneer 1921 state tax rate amounted to 2¢ per pack (of 20 cigarettes). Given that the pack price of a premium brand of cigarettes at the time was around 15¢, the resulting price increase would have been nontrivial if it were entirely shifted onto the consumer. Similar taxes were introduced across the country, typically amounting to between 10% and 15% of the existing price. A 2¢ state tax on each package of cigarettes was proposed in Minnesota in 1929 (The Wall Street Journal “Minnesota Cigarette Tax Bill Killed” 1929) and passed in Illinois in 1931, whereas proposals from the Georgia and Kentucky legislatures consisted of a 10% tax (The Wall Street Journal “Kentucky Cigarette Tax” 1930; “Tobacco Tax Upheld” 1924). A *New York Times* article more elaborately discussing the 1939 introduction of a cigarette tax in the state of New York reported retailers’ plans to absorb half the tax, increasing the consumer price of a pack of cigarettes by 6.25% (The New York Times “Cigarette Prices Under Tax to be 15c: Retailers Here Plan to Absorb Half of the New State 2-Cent Sales Levy” 1939). By 1930, an additional 12 states had followed Iowa’s example by implementing a cigarette tax; ten years later, this number had increased to 27. Figure 1 displays the spatial differences in timing.

Despite these new taxes – and the associated price increases – the rapidly increasing per capita consumption of cigarettes at the national level did not plateau until the 1960s. In parallel with aggressively promoting the use of cigarettes, the tobacco industry waged a successful information war using medical professionals to dispute the notion that tobacco was associated with any health adversity during the first half of the century (Tobacco Merchants Association of the United States *About cigarettes* 1920). In an article entitled “The Truth About Tobacco,” published in 1943, a medical doctor suggested a range of alternative explanations for substantially elevated mortality rates among heavy smokers. He proposed that those elevated rates should be attributed to the “temperament, emotional drive, (and) business worries” of the group in question (Feldt 1943). The tide eventually started turning, however, beginning with the first study conclusively demonstrating harmful health consequences of tobacco consumption in 1954 (Sloan, Smith, and Taylor, Jr. 2002), followed by a rapidly growing body of scientific evidence. The surgeon general’s comprehensive report in 1964 is typically characterized as a watershed moment in terms of a shifting public opinion regarding smoking. In subsequent years, policymakers increasingly motivated changes to tobacco taxation by the need to provide individuals with incentives not to smoke out of concerns for their health.

### 3.3 Using taxes to measure variation in cigarette consumption

Previous research has provided ample evidence of a link between cigarette taxation and rates of cigarette consumption. The mechanism through which a tax hike influences consumption is typically prices: Higher taxes typically raise consumer prices, and higher prices discourage consumption. In this paper, we follow the earlier literature, explicitly examining the consequences of early life exposure to the implementation of state-level cigarette taxation in the United States. Since we lacking individual-level data on cigarette consumption, our strategy rests on the expectation that (1) the imposed taxes were at least partly passed on to the consumer through a higher price for cigarettes and (2) this resulted in lower levels of consumption of cigarettes. As a consequence, the tax implementation reduced the prevalence of cigarette smoking, therefore reducing exposure among cohorts exposed during early life.

We first address whether it is plausible to expect that the tax was passed on to the consumer through higher prices. Based on annual data between 1955 and 2014 (Orzechowski and Walker 2014), our calculations indicate a very high correlation between state-level cigarette prices and tax rates, amounting to 0.97. While there are no systematic studies of the relationship between state taxes and the consumer price of cigarettes for the time period we examine in this paper, anecdotal evidence suggests such as relationship. For example, in a 1938 address, the dean of the University of Illinois stated, “The most immediate effect [of state taxes] is on the consumer (…). With the same expenditures he must get along with fewer cigarettes. What he formerly spent for this difference he hands over to the State” (Tobacco Merchants Association of the United States *Facts About Tobacco Taxation: Reasons Why Tobacco Products Should Not Be Taxed by States* 1941). Evidence from a more contemporary context comprehensively supports the notion that taxes on cigarettes get passed on to the consumer through increased prices. Focusing on the US context, several studies have found evidence that at least part of the tax gets passed on to the consumer (Sumner and Ward 1981), with the increase in some cases exceeding the size of the tax levied (Barzel 1976; Keeler et al. 1996; Sullivan and Dutkowsky 2012). Cheng and Kenkel (2010) conclude that state cigarette tax rates are good proxies for consumer-paid prices, with differences in tax rates accounting for much of the state-to-state differences in average prices.

Second, we address whether higher prices result in reduced smoking prevalence. From a theoretical perspective, cigarettes are peculiar goods, since the demand for them is driven (at least in part) by nicotine dependence. As a result, cigarettes are goods characterized by – at least among current users – a comparatively inelastic demand curve. The implication is that the seller should be able to pass on the tax to the consumer with fairly modest changes in demand. However, existing studies consistently show that consumption declines because of price increases. Licari and Meier (1997) examined US data for the period 1951 through 1994 and found that a 1% real increase in state cigarette taxes reduced consumption by 0.81 packs per person. These results are confirmed by Chaloupka et al. (2002), who like Powell, Tauras, and Ross (2005) also used US data to show that those most affected by price increases are younger and/or lower-income, less well-educated individuals – people whose ability to maintain their consumption would be most affected by price increases. The findings of Ringel and Evans (2001) suggest large declines in smoking rates among pregnant women in response to a tax hike. The fact that the study context is the contemporary United States should be emphasized, as these women likely represent a group with very strong incentives to quit given the widespread knowledge concerning the adverse health consequences of smoking.

**Figure 2:**
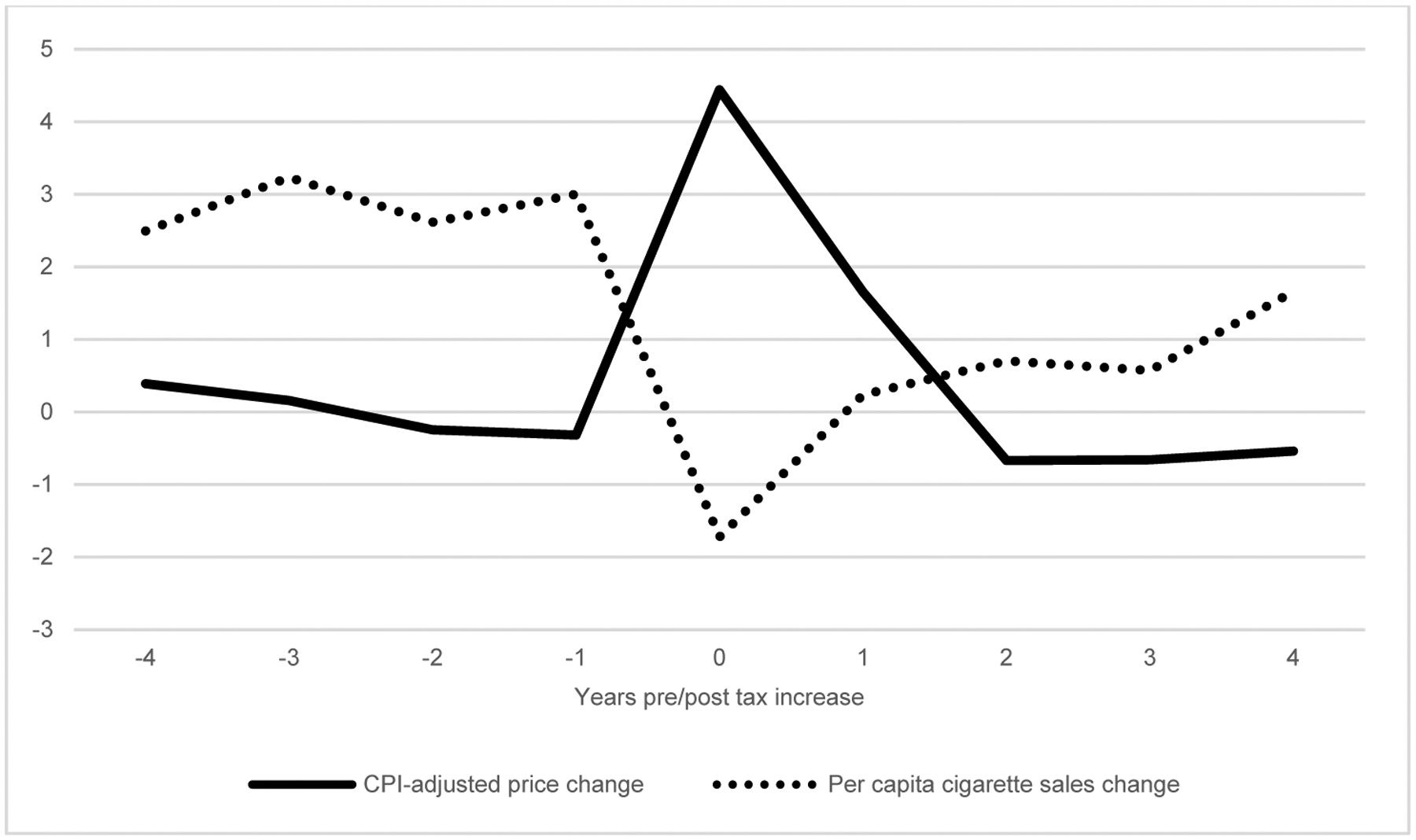
Average CPI-adjusted price change and per capita cigarette sales change (in percent) around year of cigarette tax implementation, 1955–1964 *Source*: Orzechowski and Walker 2014; own calculations.

In the time period we consider, records and reports produced by lobbying organizations paint a picture confirming that state taxes on cigarettes lead to reduced consumption (Tobacco Merchants Association of the United States *Facts About Tobacco Taxation: Reasons Why Tobacco Products Should Not Be Taxed by States* 1941). Indeed, in this era it was not uncommon for cigarette manufacturers to place newspaper advertisements offering to pay the taxes on an initial or limited-quantity purchase of cigarettes. Despite the aforementioned evidence, it is clear that there is little to suggest that the implementation of state-level cigarette taxation across the United States did much to affect overall consumption in the aggregate. Using annual data on state-level cigarette taxes, prices, and consumption for the time period 1955–1964 (Orzechowski and Walker 2014), we investigate responses to the implementation of state-level taxes during a period that should be highly similar to the one examined in this paper. Most importantly, due to the period occurring prior to the surgeon general’s report on the negative health effects of smoking, individuals’ reactions to a price increase in terms of their consumption behavior should have been similar. Figure 2 shows the state-level average annual change in (1) inflation-adjusted price and (2) per capita sales during years surrounding tax increases. The time-series is centered at the year of a tax change, clearly showing how the change in price during the years prior to implementation of the tax increase follows inflation. During the year of implementation of a change in the state tax on cigarettes, the change in the price of cigarettes outpaces inflation by – on average – 4.4%. The year after implementation of the tax increase also displays a price change exceeding the pace of inflation, suggesting that in some cases the date of implementation occurred during the latter part of the year. Subsequently, the annual change in cigarette prices again returned to follow inflation.

Turning to the annual change in per capita cigarette sales, Figure 2 indicates a steady rate of increase of between 2% and 3%. More importantly, the figure illustrates how the tax-induced price increase had an immediate influence on consumption, on average reducing the per capita sales of cigarettes by 1.7% compared to the year prior to the tax increase. The short-term nature of the reduction in consumption also emerges from the figure, with the per capita growth in sales already increasing in the year after the tax change. In conclusion, provided that the same dynamics applied during the period we examine in this paper, we can expect reductions in cigarette consumption particularly during the year of implementation of state cigarette taxes; consumption thereafter gradually increased to pre-implementation levels within a few years.

### 3.4 Hypotheses

Based on the previous section, we formulate the following hypotheses:

*Hypothesis 1:* The implementation of state cigarette taxes caused a one-time increase in prices, resulting in a reduction in consumption. People who were in utero or in infancy at the time a cigarette tax was first introduced in their state would therefore have been less exposed to cigarette smoke than surrounding cohorts, resulting in an older age at death.

We also expect certain groups’ smoking behavior to have been more strongly affected by the implementation of state-level cigarette taxation. To the extent that our expectations are confirmed, this provides further support that observed associations reflect that this changing behavior was directly responsible for variations in observed life span. Firstly, as noted above, smoking was more common in urban areas, primarily driven by a pronounced difference in smoking prevalence between individuals engaged in farm and non-farm occupations. In addition to farmers less frequently being smokers, in this era in which smoking was nearly universally allowed in homes, in public, and in workplaces, exposure to secondhand smoke would generally have been lower for households engaged in farming activities. Consequently, we hypothesize that exposed children in households engaged in occupations outside farming would have been subjected to the most pronounced reduction in exposure to smoking, translating to the largest gains in terms of age at death.

*Hypothesis 2:* The age at death of children of rural residents – and farmers in particular – was least affected by the implementation of state cigarette taxation due to the lower baseline exposure to smoking in this group. Conversely, urban, non-farm residents would have experienced the most substantial reduction in exposure and therefore also the largest gain in terms of the age at death.

Across states implementing cigarette taxes, the purchase of cigarettes was frequently also subject to different age eligibility restrictions. While this did not necessarily prevent minors from acquiring cigarettes or from becoming exposed, it would nevertheless have increased the level of difficulty in doing so. An association that is observed only among children of mothers who were of age to purchase cigarettes is thus consistent with the behavior of individuals in this group, since it was the only one affected.

*Hypothesis 3*: The consumption of cigarettes by (expectant) mothers whose age made them ineligible for purchasing cigarettes should have been relatively unaffected by the implementation of state cigarette taxes. Consequently, the effect of cigarette taxes on the age at death of children with mothers whose young age prevented them from buying cigarettes should be trivial compared to the effect on children with age-eligible mothers.

Individuals who lived in states that implemented a cigarette tax but lived in close proximity to a state that had yet to implement such a tax would have had greater ability to purchase cigarettes across the state border. Indeed, the effect of state taxes on cigarette vendors that operated close to state borders was a subject of great concern for the tobacco lobby at the time. It claimed that this situation resulted in the loss of business since “consumers (…) cross the border and make their purchases (…) not alone for themselves but for their neighbors and friends as well” (Tobacco Merchants Association of the United States *Facts About Tobacco Taxation: Reasons Why Tobacco Products Should Not Be Taxed by States* 1941). Another possibility, but one with largely the same outcome, is that vendors that operated close to a state border might have opted to shift a smaller share of the tax to consumers, resulting in cheaper cigarettes than in locations characterized by a greater distance to a neighboring state without cigarette taxes.

*Hypothesis 4:* The effect of state cigarette taxes on cigarette consumption is greatest among women who lived far from borders with states that had yet to implement cigarette taxes. As a consequence, the greatest gains in age at death will be observed among children of said mothers.

## 4. Data and methods

For the analysis, we combined several sources of data. Information on the year of implementation of state cigarette taxes was obtained from Orzechowski and Walker (2014). While there should be little doubt that the introduction of state taxes on cigarettes increased prices and thereby the cost of maintaining an unchanged habit, it is equally true that taxes did not halt the overall trend toward increasing cigarette consumption. Consequently, whatever influences state taxes had on consumption at the state level, they were likely to have been relatively short-lived, consistent with the scenario illustrated in Figure 2.

To consider how early life exposure to the implementation of a state-level cigarette tax – and thus a presumed reduction in cigarette smoking – influences age at death, we use individual-level data obtained from the full-count US censuses of 1930 and 1940 (Ruggles et al. 2019), combined with death records from the US Social Security Death Master File (SSDMF)^8^ merged with Social Security claims data from the Numident (Breen and Goldstein 2022).^9^ The baseline sample includes all males born in the United States between 1920 and 1940 who were the children of their household head.^10^ From the censuses, we further exploit a range of individual- and household-level characteristics that potentially moderate or mediate the association between early life exposure to state-level cigarette taxation and age at death. We control for boys’ socioeconomic background through the occupational score of the father, in addition to the race of the individual (white/nonwhite), the age of the mother at the child’s birth, whether the individual resided on a farm, and whether he lived in a rural or urban area. Lastly, each boy’s state and year of birth captures time-invariant differences in, for example, life expectancy, smoking habits, and exposure to other pollutants.

**Figure A-1:**
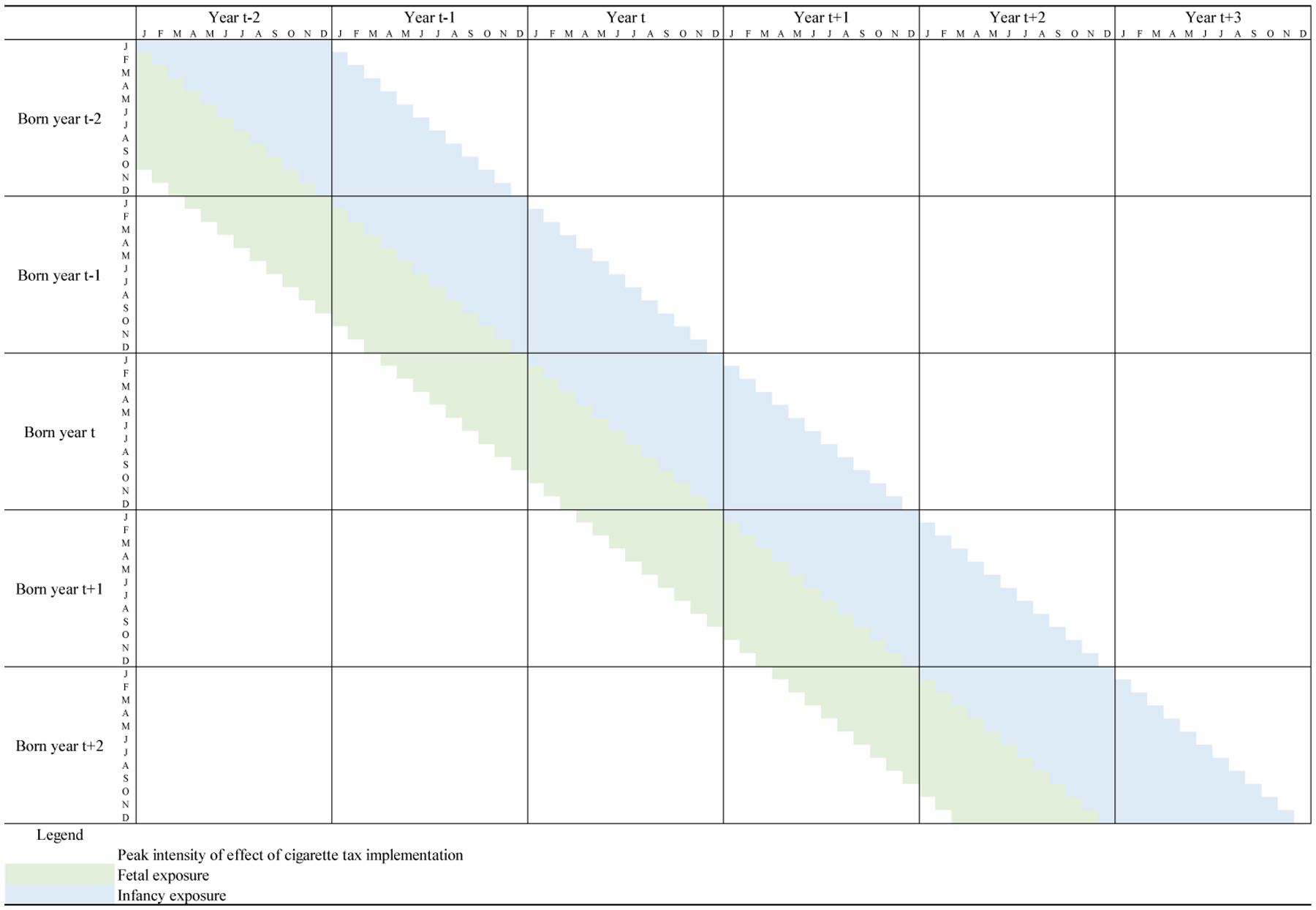
Exposure to peak intensity of effect of cigarette tax implementation by calendar time (horizontal axis) and cohort (vertical axis)

Information on age at death is obtained from death records, through dates of birth and death,^11^ measured in fractions of a year. Date of birth is also used to determine early life exposure to the implementation of cigarette taxes, which we base on information on the year of the tax implementation, illustrated by Figure A-1 in Appendix A. Following the horizontal axis, displaying calendar time, we define the key period for the consequence of the implementation of the cigarette tax to occur during the year of implementation, as illustrated by the shaded area. While sporadic data at our disposal confirms the implementation of the tax occurring at various times throughout the year, we also demonstrate that the most pronounced decline in consumption appears to have occurred during a period limited to the year of implementation. We define individuals born either during the year of implementation or the year after as those maximally exposed during the fetal stage or infancy. More specifically, following the assumption that the peak period of exposure is limited to the year of implementation, Figure A-1 illustrates how the majorities of both cohorts are exposed during the fetal stage, with the cohort born during the year of implementation also experiencing exposure during infancy. While admittedly an imperfect definition, this nevertheless isolates the birth cohorts most exposed during this key stage of physiological development.

### 4.1 Record linkage

We initially extracted the full cohorts of US-born men born between 1920 and 1929 from the 1930 full-count census and those born between 1930 and 1940 from the 1940 census, conditional on their being a son of the household head. The resulting baseline populations, amounting to 10 million and 9 million boys, respectively, were subsequently linked to their death records using probabilistic methods of record linkage. Death records were obtained through the SSDMF, which contains roughly 93 million unique deaths occurring between 1899 and 2013. To about a third of the deaths recorded in the SSDMF we have added information from the Numident claims file, providing additional valuable information for record linkage,^12^ including parents’ names, sex, and state of birth. Using the SSDMF also motivates restricting the study population to males, as this source does not allow us to distinguish women’s married names from their names at birth. Consequently, relying on names reported in the SSDMF and linking to census records from when an individual was a child would have introduced a substantial amount of linking error.

Our study sample is followed from birth until age 73 (93) for the youngest (oldest) birth cohort included. From the late 1970s until the early 2000s, almost all deaths are covered in the data, but the periods before and after are characterized by substantial under-coverage of deaths. As a result, our data in practice fully cover only deaths occurring in the age interval 35–73 for the youngest cohort and 55–93 for the oldest.

We link records across our sources of data based on the probit machine learning algorithm proposed by Feigenbaum (2016). For these purposes, we rely on the record linkage software Hlink (Wellington, Harper, and Thompson 2022), developed at the Minnesota Population Center, in combination with a refined version of the Feigenbaum algorithm. The refinement refers to the use of – when available – a broader set of observable characteristics used by the algorithm to assess matches, including name similarity scores for the mother and father’s first name, as recommended by several recent record linkage efforts (Bailey et al. 2022; Helgertz et al. 2022).

We train the algorithm to optimize its performance according to the Matthews correlation coefficient (MCC), designed to work particularly well for unbalanced data (i.e., where there is a very small share of matches) (Chicco and Jurman 2020). This is performed following a train-test-split procedure, where the data are randomly split into two equally sized parts, followed by the algorithm being trained on one-half of the data and its performance being evaluated on the other half, for which the predicted matches can be compared to those designated as true. As the training data are a random subset of the data we aim to link, the performance statistics should reflect that in the larger dataset as well.

The optimized probit algorithm is subsequently applied to the full data, using thresholds uniquely calibrated for each respective set of data. For both the 1930 and the 1940 census populations, we primarily rely on links made to the Numident data, due to its greater precision, which is connected to the comparative richness of linking information. For 1930 and 1940 census individuals who were not linked to the Numident, we complement with links obtained through the SSDMF. After completing this step and completing additional steps of data cleaning,^13^ we are left with 2.4 million successfully linked boys.

To investigate the robustness of our main results, we conduct supplementary analyses relying on publicly available sources of linked data that also allow us to examine the mortality of outlined cohorts, namely Censoc links between the Numident/SSDMF and the 1940 census (Goldstein et al. 2021), the Census Linking Project (Abramitzky et al. 2020), and Multigenerational Longitudinal Panel (Helgertz et al. 2020) census-to-census links. These analyses and the results thereof are more comprehensively described in Appendix B, illustrating that our key results are unlikely to be driven by the choice of linking method.

### 4.2 Empirical strategy

The baseline analysis is performed following Equation (1), estimated by means of OLS regression:

(1) $$Y_{ist}=\gamma_{s}+\mu_{t}+\beta D_{st}+\rho X_{i}+\varepsilon_{ist}$$

Y_ist_ refers to the age at death of individual *i*, born in state *s* at time *t*. γs represents state-of-birth fixed effects, with μt representing birth cohort fixed effects. State-of-birth fixed effects are important, as they account for unique characteristics at the state level, such as baseline differences in tobacco use as well as between-state differences in disease load, school quality, level of economic development, and so on. The year-of-birth fixed effects aim to account for changes in life expectancy due to secular improvements in living standards, environmental shocks occurring during the time period, and those that may have affected birth cohorts in particular ways (e.g., the Great Depression and the Dust Bowl). In our preferred specifications, we opt against interacting the year and state-of-birth fixed effects due to the small number of resulting observations in certain cells and concerns about overfitting the model. Additionally, our results are robust to controlling for early life exposure to the Dust Bowl. The parameter of interest, βDst, captures the effect of the introduction of cigarette taxation, taking the value 1 for cohort *t* born during the year of or the year after the tax implementation in state of birth *s* and taking the value 0 otherwise. The X vector includes the aforementioned sociodemographic and geographic control variables, including the individual’s parents’ socioeconomic status. Lastly, εist is an individual specific error term. To investigate outlined hypotheses, the effect of early life exposure to state cigarette tax implementation will be allowed to differ according to several of the variables included in the X vector.

To account for the possibility that our study sample does not reflect the underlying population due to selection in linking, our models are estimated using post-stratification weights^14^ (Bailey, Cole, and Massey 2020). These are generated to make the study populations extracted from the 1930 and 1940 censuses resemble their baseline populations in terms of year of birth, region of birth, and race. Models are estimated using Stata version 16, with standard errors clustered at the state-of-birth level. In addition to our main models, we furthermore conduct a selection of robustness checks, including lead-lag models and models with placebo exposure to cigarette taxation.

### 4.3 Descriptive statistics

Unweighted sociodemographic characteristics of the analytical sample, consisting of 2.4 million men, are displayed in Table 1. For all individuals to be observed in an identical age-at-death interval, we restrict the sample to individuals dying between the ages of 55 and 73, with the mean age at death being 65.7. While this excludes a substantial number of successfully linked individuals who died at younger and older ages, we nevertheless opt for this restriction for the main model specification to avoid introducing systematic differences in age at death resulting from certain cohorts’ age-at-death window differing from that of others. This also results in the population lining up rather well with the estimated life expectancy at birth according to the Social Security Administration: 61.8 for the 1920 cohort and 69.6 for the 1940 cohort (Bell and Miller 2005).^15^

The study population is distributed evenly across the included birth cohorts, with 5.6% – or about 135,000 individuals – considered to have been exposed to the peak reduction in cigarette smoking resulting from the implementation of state taxes when they were in utero and in infancy. Control variables measure a range of sociodemographic and geographic characteristics, relevant in their own right and when testing for hypothesized heterogeneities in the effects of fetal exposure to state cigarette taxes on age at death. The table illustrates how roughly 30% of boys resided on a farm in the first census in which they were enumerated, with an overall slight dominance of rural residents. Our models also control for mother’s age at the time of the individual’s birth. While we do not expect individuals with very young or very old mothers to die at younger ages, we do expect a stronger response among individuals with mothers who were legally prohibited from purchasing cigarettes in their state of residence.^16^ The sample additionally displays a distribution by race and origin socioeconomic status that corresponds rather well to the underlying baseline populations.^17^

Lastly, the data indicate that the average individual was born in a county that was 94 miles (Euclidian distance) from the nearest adjacent state that implemented a cigarette tax later than the individual’s own state of birth. Displaying a considerable skewness toward shorter distances, this information will be used to examine whether the sensitivity to a state tax on cigarettes was greater for individuals with fewer possibilities to access alternative markets in a neighboring state.

## 5. Results

Our main results from OLS regression models are presented in Table 2.^18^ Apart from the effect of early life exposure to the peak reduction in smoking due to the implementation of state-level cigarette taxation, Model 1 includes only year and state-of-birth fixed effects. Net of these fixed effects, the model suggests that being exposed to the implementation of state-level cigarette taxes during early life translates to an increase in age at death of 1.5 months (b = 0.125, s.e. = 0.055) compared to surrounding cohorts.^19^ In Model 2, the full set of individual and contextual controls is added; the association is essentially unchanged. Lastly, the estimate represents an average treatment effect rather than a treatment effect on the treated. More specifically, the estimate represents a population average, not accounting for the fact that only a marginal share of the population would have been affected to any considerable extent by the cigarette tax. As a consequence, our estimates are likely to underestimate the effect among those whose exposure to the consequences of the implementation of the tax was the greatest.

In an attempt to better grasp such heterogeneities, we proceed to estimate models where the effect of early life exposure to the state tax on cigarettes is allowed to vary across groups, whose intensity of exposure is theoretically motivated to differ. We begin with Model 3, investigating whether patterns of the effect of fetal exposure to cigarette taxation are consistent with known patterns in overall use and exposure at the time. As previously outlined, cigarette smoking during this era was much more prevalent among the urban, non-farm population; for this reason, we expect to see greater gains in the age at death within this population through a larger response from the tax implementation. The results are fully consistent with the expectations, suggesting no effect among children of farm dwellers. Among the rural farm population, the point estimate suggests a marginal 0.6-month-later age at death among those exposed during early life (β = 0.05, s.e. = 0.07)) compared to surrounding birth cohorts who were not exposed to the tax implementation during this key phase of development. While the estimate for farm dwellers in areas classified as urban is considerable, it is estimated without precision as well as being based on too small a sample (N = 4,800) to warrant meaningful interpretation. Turning to the estimates for the non-farm dwellers, they indicate a two-month-later age at death compared to surrounding birth cohorts, regardless of whether the place of residence is urban or rural.

Model 4 proceeds to investigate whether children of women who were below the legal age for purchasing tobacco at the time of the cigarette tax implementation experienced less of a gain in age at death than children of women without exposure to such a restriction. While such legislation would not have completely prevented individuals under the legal age from acquiring cigarettes, it would have made being successful in this endeavor more difficult. The slight reduction in sample size, due to incomplete information on the age limit in some states, should here be noted. Additionally, since the data indicate only the age range of the age restriction,^20^ we estimate separate models imposing different variants of the age restriction. Model 4a imposes age restrictions of 17, 20, and 23 years, depending on the state category in which the individual was born, whereas the corresponding ages for the less strict age restrictions of Model 4b are 15, 18, and 21 years of age. Across both models, the estimates indicate a trivial effect from early life exposure to state-level cigarette taxation among children whose mothers were below the age threshold for being eligible to purchase cigarettes at the time of the tax implementation. Additionally, the point estimates are smaller (β = 0.09, s.e. = 0.09 [4a], β = 0.05, s.e. = 0.098) [4b]), consistent with the hypothesis that the expected increase in cigarette price did little to change behavior, as the mothers were unlikely to have been cigarette smokers. In contrast, children of both younger and older mothers – but whose mothers were above the legal age for purchasing cigarettes – are observed with larger effects, indicating that the reduction in early life exposure to cigarette smoking translated to an older age at death compared to surrounding cohorts, amounting to between 1.6 and 2.4 months, respectively.

We lastly proceed to Model 5, examining whether having plausible access to an alternative market for purchasing cigarettes influenced how households were affected by state cigarette taxes. For this purpose, we limit the sample to individuals born in states that were adjacent to at least one state that implemented the cigarette tax at a later point in time. The expectation is that the farther the distance to the border, the greater the effect of the tax, as the consequences would be more strongly felt in areas with fewer possibilities to access alternative markets for cigarettes. This would result from greater opportunities to avoid the tax through purchasing cigarettes across the border, something that would also provide cigarette vendors with greater incentives to absorb a greater share of the imposed tax. The preferred specification suggests a rather strict threshold in terms of the distance to the nearest neighboring state,^21^ with no effect from the cigarette tax at distances up to 15 miles (N = 115,000) and with a consistent gain in age at death among individuals at greater distances. More specifically, the model indicates a marginal effect among children exposed in early life residing in close proximity to a state that had yet to implement a tax on cigarettes, translating to a 0.8-month-older age at death (β = 0.07, s.e. = 0.096).^22^ In contrast, and consistent with our hypothesis, those residing at a distance greater than 15 miles experienced a greater increase in the predicted age at death if exposed to the peak effect of state-level cigarette taxation during early life, amounting to 1.6 months (β = 0.13, s.e. = 0.05).

## 6. Robustness analysis

The results have told a story that is consistent with theoretical expectations regarding groups whose consumption would have been most affected by the price increase resulting from the implementation of state-level cigarette taxes, thereby also translating to the greatest gains in age at death. To strengthen our confidence in the validity of our results, we have replicated our main specifications using three other publicly available linked datasets: the Censoc (Goldstein et al. 2021), the Census Linking Project (Abramitzky et al. 2020), and IPUMS MLP (Helgertz et al. 2020). As shown in Appendix B, our results are quantitatively and qualitatively similar. Consequently, our results are not driven by a process unique to the linking algorithm selected for this paper. Another concern relates to the inherent uncertainty concerning the timing and duration of exposure. Without information on the exact date state taxes went into effect and being able to only approximate how long cigarette consumption declined as a result of those taxes, accurately isolating the precise birth cohorts of exposure is problematic. In addition, while our focus is on the consequences of early life exposure to maternal smoking, defined as fetal and infancy exposure, we also know that the first few years of life remain essential for human lung development. Consequently, while we expect the reduction in cigarette consumption to have been short-lived, the benefits may have extended to individuals exposed past infancy.

**Figure 3:**
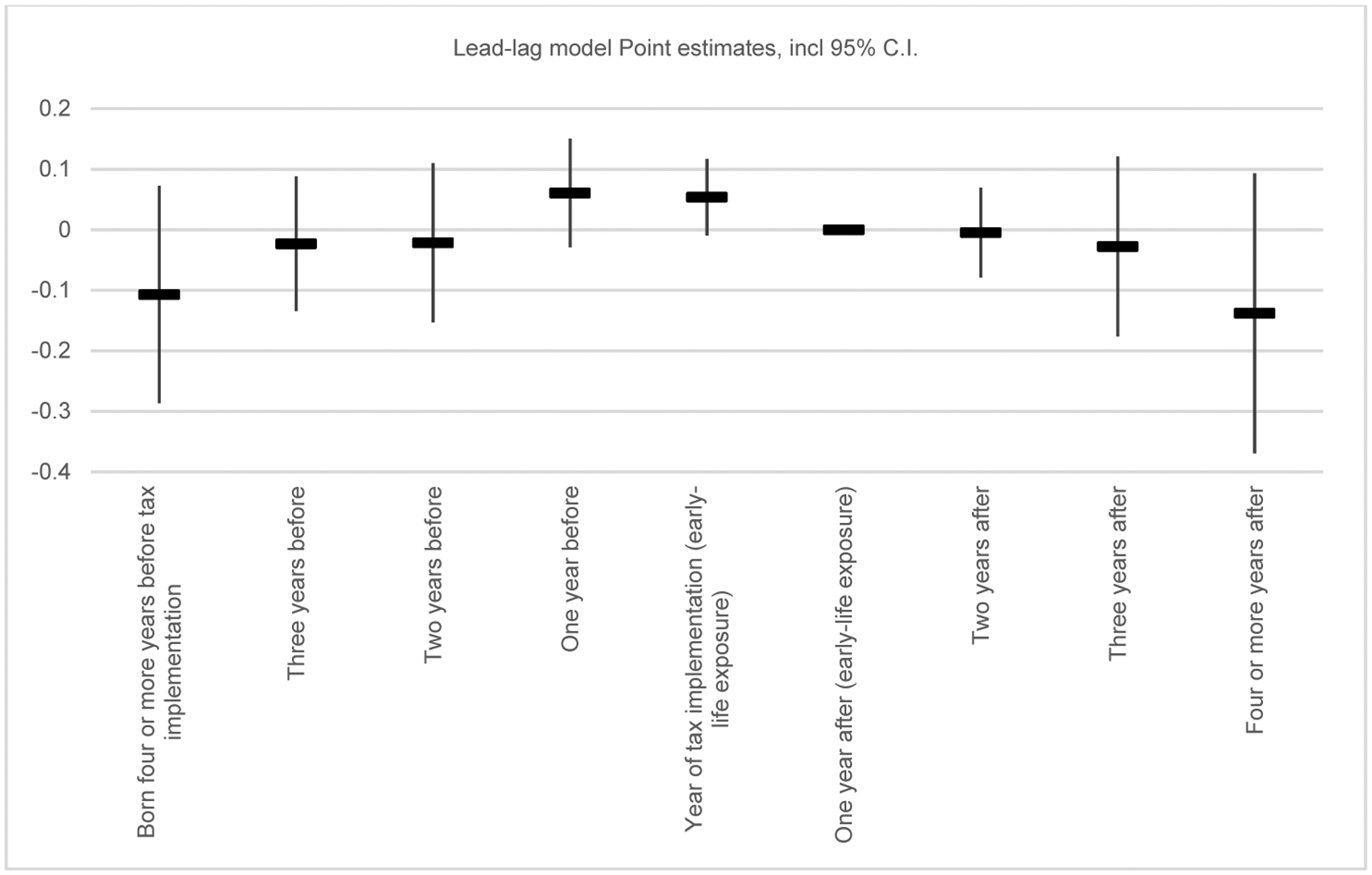
Lead-lag model estimates

We therefore estimate a model allowing for full flexibility for the years of birth immediately before and after state cigarette tax implementation. The reference category is represented by individuals born the year after implementation, who thus belong to one of the early life cohorts from the main analysis. The results are shown in Figure 3, illustrating a scenario consistent with a short-term reduction in smoking because of the cigarette tax, as well as benefits that are greatest for those exposed while in utero or during the first two years of life. While the coefficients are not estimated with enough precision to be significantly different from each other, the negative point estimates for those born two or more years before implementation are consistent with these cohorts having completed the key phase of lung development by the time of the tax implementation, in time to enjoy the health benefits linked to a reduction in consumption. Relatedly, the lack of any health benefits experienced by those born a few years after the tax implementation is consistent with cigarette consumption returning to (and eventually exceeding) pretax levels within a few years.

We lastly estimate the effect of early life exposure to state-level cigarette taxation using brother fixed effect models, resulting in a smaller sample (N = 249,000) as the estimator relies on within-family variation. While the smaller sample size represents a disadvantage, the ability to obtain estimates net of all observed and unobserved characteristics that are constant and shared between the brothers is a major strength. The results are presented in Table A-2 and illustrate that the age at death of the brother exposed during early life to a reduction in exposure to cigarette smoke brought about by state taxation on average is 2.2 months higher (b = 0.186) than that of the unexposed brother, further reinforcing the validity of our results.

## 7. Discussion

Smoking is not only the main underlying determinant of preventable death among adults in contemporary developed countries; it also remains a frequent early life exposure in spite of the negative health consequences being both thoroughly demonstrated and well-known. Despite the plethora of research into the association between being subjected to the many harmful substances of cigarette smoke and health outcomes, research on human populations remains predominantly associational as well as focused on outcomes observed rather soon after exposure. We exploit a new data source developed for the purpose of examining the link between early life exposure to an exogenous reduction in cigarette smoking and individuals’ ages at death. Using state-of-the-art methods of record linkage, we link full-count census data to death records, thereby being able to investigate the link between early life exposure and the implementation of a cigarette tax at the US state level for cohorts of boys born between 1920 and 1940.

Our results show that individuals who were exposed to the implementation of a state tax on cigarettes in utero or during infancy enjoyed a nontrivial delay of age at death, amounting to 1.7 months. While not remarkable in size, the effect is larger than the difference in age at death between children of fathers belonging to the highest and lowest occupational score groups. While the individual’s own attained socioeconomic status most likely is a more important determinant of age at death (Halpern-Manners et al. 2020), socioeconomic origin remains an important independent factor, shown in other research to influence the risk of the onset of certain conditions later in life through the individual being subjected to deprivation in childhood (Smith et al. 2003). Furthermore, given the well-established and strong relationship between social class and health today – although its historical permanence appears to be increasingly unclear (Bengtsson, Dribe, and Helgertz 2020) – we believe this further promotes the importance of our results.

Our results should also be interpreted against a backdrop where smoking prevalence was still on the rise and far from the levels it would reach a few decades after the end of the study period. This particularly applied to women, implying that the most common type of exposure would have been in the form of secondhand smoking. Consequently, the average treatment effect estimated in our analysis may be considerable underestimation of the longevity benefit enjoyed by children of mothers who were smokers themselves. In fact, should historical responses to tax hikes resemble those in the contemporary United States, as reported by Evans and Ringel (1999), this would indeed be the case.

While our linkage rate is by no means unremarkable in comparison with related efforts to link data like these, the sample analyzed is nonetheless unlikely to be a representative subsample of the underlying population. This pertains not only to the fact that we examine only boys but also concerns which boys are ultimately linked across census years. Caution is therefore warranted, particularly relating to the extent the conclusions extend to underrepresented subpopulations, including the African American population, not to mention the population of girls. Another limitation concerns only observing deaths in the age interval 55–73, introducing two separate selection mechanisms – the net consequences of which are difficult to fully evaluate. While the lower age restriction introduces positive selection on health, the upper restriction has the opposite effect on the sample. This is an important limitation and should be borne in mind when interpreting the results. For example, it is unfortunate that we are unable to translate our results to a more straightforwardly interpretable measure, such as life expectancy at birth. Lastly, several key factors are measured with considerable imprecision. Most notably, this concerns both the timing of the tax implementation and the beginning and end of the in utero and infancy periods.

Despite aforementioned limitations, our confidence in our results is reinforced by the results of our comprehensive effect heterogeneity analyses. More specifically, those results show substantially attenuated (or null) associations among subpopulations we hypothesized to have been the least exposed to the consequences of the tax implementation, including individuals whose parents resided on a farm, who lived in close vicinity to a neighboring state without a tax, or whose mother was below the legal age for cigarette purchase. The results suggest that a typical difference in life expectancy between the groups enjoying the largest benefits from the implementation of a cigarette tax and the groups that were less affected amounts to up to two months. It also deserves to be mentioned that the interactions are robust to a range of alternative specifications.

Some members of the cohorts we examined were subjected to a modest and temporary decrease in exposure to smoking as a result of a state cigarette tax, after which they were subjected to several decades of postnatal exposure to an environment characterized by an increasing prevalence of smoking in virtually all social contexts. More specifically, not until the 1964 report of the surgeon general did the tide start to turn in the war against tobacco and the gradual implementation of smoking bans and additional – and more dramatic – tax increases to disincentivize cigarette consumption (Cole and Fiore 2014). Considering this environment, the longevity consequences of the introduction of the first state cigarette taxes likely represented a nontrivial contribution to life expectancy gains being made during this period. Unfortunately, it is beyond the scope of this study to address whether the observed relationships also apply to girls, with them being less vulnerable during the fetal stage than boys. This should be the topic of a future study using similar data.

## Supplementary Material

Data info

## Figures and Tables

**Table 1:** Descriptive statistics

	Mean	Min	Max	s.d.
Age at death	65.7	55	73.0	4.8
Year of birth	1929.3	1920	1940	5.8
Early life exposure to state cigarette taxation	5.6	-	-	-
Mother’s age at individual’s birth	27.1	13	45	6.6
Father’s occupational score:				
0‒20	49.6	-	-	-
21‒40	38.3	-	-	-
41‒80	7.0	-	-	-
N/A	5.2	-	-	-
Race:				
White	89.6	-	-	-
Nonwhite	10.4	-	-	-
Individual’s place of residence:				
Urban, non-farm	47.4	-	-	-
Urban, farm	0.2	-	-	-
Rural, non-farm	22.9	-	-	-
Rural, farm	29.5	-	-	-
Distance to nearest county in adjacent non-cigarette-tax state (miles)*	93.6	5.1	716.1	111.3
Individuals (observations)	2,419,343			

* Limited to individuals born in states that, at time of birth, neighbored at least one state that had yet to implement cigarette taxation (N = 1,828,132)

**Table 2:** OLS regression estimates

	Model 1	Model 2	Model 3	Model 4a	Model 4b	Model 5
Early life exposure (state cigarette tax implemented during year of birth or year after birth)	0.125** (0.0551)	0.124** (0.0550)				
Early life exposure, urban non-farm			0.166*** (0.0555)			
Early life exposure, urban farm			−0.494 (0.312)			
Early life exposure, rural non-farm			0.163*** (0.0591)			
Early life exposure, rural farm			0.0516 (0.0698)			
Early life exposure; mother below legal age for cigarette purchase				0.0924 (0.0978)	0.0516 (0.0979)	
Early life exposure; mother age </= 23 and eligible for cigarette purchase				0.199*** (0.0586)		
Early life exposure; mother age > 23 and eligible for cigarette purchase				0.132** (0.0618)		
Early life exposure; mother age </= 21 and eligible for cigarette purchase					0.192** (0.0717)	
Early life exposure; mother age > 21 and eligible for cigarette purchase					0.135** (0.0613)	
Early life exposure; distance to state without cigarette tax </= 15 miles						0.0692 (0.0962)
Early life exposure; distance to state without cigarette tax > 15 miles						0.133*** (0.0498)
Constant	65.66*** (0.0335)	65.52*** (0.0468)	65.52*** (0.0462)	65.53*** (0.0496)	65.51*** (0.0510)	65.53*** (0.0527)
State-of-birth FE	Y	Y	Y	Y	Y	Y
Year-of-birth FE	Y	Y	Y	Y	Y	Y
Father’s occupational score	N	Y	Y	Y	Y	Y
Place/type of residence	N	Y	Y	Y	Y	Y
Race	N	Y	Y	Y	Y	Y
Mother’s age at individual’s birth	N	Y	Y	Y	Y	Y
Observations	2,419,343	2,419,343	2,419,343	2,199,294	2,199,294	1,828,832
R-squared	0.021	0.021	0.021	0.021	0.021	0.021

*Notes*: Robust standard errors in parentheses.

* p < 0.1,

** p < 0.05,

*** p < 0.01.

Model 5 is restricted to individuals born in states for which we have information on age restriction on cigarette purchases.

Model 6 is restricted to individuals residing in states having at least one neighboring state that had yet to implement cigarette taxation.

## Footnotes

3 At least two publications authored by scientists in Germany during the Second World War are today sometimes acknowledged as having provided the first empirical evidence linking smoking with cancer (Hermann 1940; Schairer and Schöniger 1944).

4 Most existing research focuses on the consequences of firsthand smoking. The few studies that examine the effects of exclusively being exposed to secondhand smoking suggest it being a significant, albeit less pronounced, exposure (Khader et al. 2011).

5 Figures reported by Milmore and Conover (1956) suggest that the annual consumption of cigarettes per person aged 14 years or above increased from 1.89 pounds in 1920 to 5.16 pounds in 1940, which translates to 1.8 ([1.89*338] (from Table 2)/365.25) cigarettes daily in 1920 and 4.9 cigarettes daily in 1940 ([5.16*348] (from Table 2)/365.25).

6 The exception being the group labeled “unable to work” – largely older individuals.

7 We are unable to account for taxation at the city/municipal level, which was uncommon.

8 The SSDMF contains more than 94 million records. The file is created from internal Social Security Administration records of deceased persons possessing Social Security numbers whose deaths were reported to Social Security.

9 The Numident contains all interactions individuals have with the Social Security Administration related to Social Security numbers, including applications, claim records, death information, and requested changes to Social Security information.

10 From the 1930 census, we extracted individuals born between 1919 and 1930; from the 1940 census, we extracted individuals born between 1930 and 1940.

11 When the day of death or birth is missing, we arbitrarily set this to the 15^th^ day of the month.

12 The number of deaths linked to the Numident claims file data amounts to only 35 million, occurring between 1900 and 2007. The advantage of relying on this source, however, pertains to the additional information available there, increasing the ability to obtain accurate links.

13 The most significant steps are restricting the individuals to those dying between age 55 and 73, as well as removing duplicate matches – i.e., mortality records linked to more than one 1930/1940 census individual. Prior to these steps, the linked sample contained 5 million boys, translating to a de facto linkage rate of 26%.

14 The post-stratification weights were generated using the ipfweight package in Stata, with the maximum number of iterations set to 100.

15 While roughly 2 million individuals are dropped from the sample due to the age-at-death restriction, not imposing this restriction yields an average age at death of the 1920 (1940) cohort of 73.8 (58.1) years, well above (below) that suggested by the life table estimates. We thus consider the restrictions as a necessity to more accurately represent the cohorts of study.

16 For this purpose, we use time-series data on age restrictions for the purchase of tobacco (Apollonio and Glantz 2016)

17 Elaborate variable descriptions are available at https://usa.ipums.org/usa-action/variables/group.

18 Full regression output is provided in Table A-1.

19 The point estimate of 0.125 implies an increased life expectancy of 13% of a year, on average lasting 365.25 days. This translates to (365.25*0.125) = 45.7 days, or 1.5 months (~45.7/30.4).

20 The categories are < 18, 18–20, and > 20.

21 A number of alternative specifications with other thresholds have been estimated, consistently indicating no effect at distances up to 15 miles and with a consistent effect at greater distances.

22 These results hold when, in an attempt to ascertain that the county of residence at that point is the same as that at the time of birth, we restrict the sample to individuals who in the 1930 and 1940 censuses resided in the state in which they were born.

## Appendix A

**Table A-1:** Full OLS regression output, main analysis

	Model 1	Model 2	Model 3	Model 4a	Model 4b	Model 5
Early life exposure (state cigarette tax implemented during year of birth or year after birth)	0.125** (0.0551)	0.124** (0.0550)				
Early life exposure, urban non-farm			0.166*** (0.0555)			
Early life exposure, urban farm			−0.494 (0.312)			
Early life exposure, rural non-farm			0.163*** (0.0591)			
Early life exposure, rural farm			0.0516 (0.0698)			
Early life exposure; mother below legal age for cigarette purchase				0.0924 (0.0978)	0.0516 (0.0979)	
Early life exposure; mother age </= 23 and eligible for cigarette purchase				0.199*** (0.0586)		
Early life exposure; mother age > 23 and eligible for cigarette purchase				0.132** (0.0618)		
Early life exposure; mother age </= 21 and eligible for cigarette purchase					0.192** (0.0717)	
Early life exposure; mother age > 21 and eligible for cigarette purchase					0.135** (0.0613)	
Early life exposure; distance to state without cigarette tax </= 15 miles						0.0692 (0.0962)
Early life exposure; distance to state without cigarette tax > 15 miles						0.133*** (0.0498)
Year of birth: 1920	ref	ref	ref	ref	ref	ref
1921	−0.0606** (0.0234)	−0.0590** (0.0234)	−0.0588** (0.0234)	−0.0589** (0.0252)	−0.0588** (0.0252)	−0.0805** (0.0326)
1922	−0.0535** (0.0211)	−0.0521** (0.0212)	−0.0518** (0.0212)	−0.0697*** (0.0245)	−0.0697*** (0.0245)	−0.0506* (0.0259)
1923	−0.00856 (0.0215)	−0.00701 (0.0216)	−0.00589 (0.0216)	−0.0107 (0.0235)	−0.0107 (0.0235)	−0.0280 (0.0238)
1924	0.000 (0.0243)	0.00147 (0.0242)	0.00260 (0.0243)	−0.00521 (0.0259)	−0.00513 (0.0259)	−0.0133 (0.0270)
1925	0.0251 (0.0215)	0.0269 (0.0215)	0.0282 (0.0214)	0.00703 (0.0209)	0.00708 (0.0209)	0.00805 (0.0286)
1926	0.0979*** (0.0231)	0.0993*** (0.0229)	0.101*** (0.0226)	0.0965*** (0.0254)	0.0964*** (0.0255)	0.0960*** (0.0280)
1927	0.159*** (0.0297)	0.160*** (0.0297)	0.161*** (0.0296)	0.150*** (0.0326)	0.150*** (0.0328)	0.156*** (0.0365)
1928	0.269*** (0.0311)	0.270*** (0.0312)	0.271*** (0.0312)	0.260*** (0.0333)	0.260*** (0.0334)	0.269*** (0.0381)
1929	0.264*** (0.0395)	0.267*** (0.0395)	0.267*** (0.0396)	0.265*** (0.0417)	0.265*** (0.0417)	0.276*** (0.0407)
1930	0.432*** (0.0411)	0.433*** (0.0411)	0.434*** (0.0409)	0.424*** (0.0446)	0.424*** (0.0446)	0.436*** (0.0512)
1931	0.485*** (0.0419)	0.486*** (0.0419)	0.486*** (0.0420)	0.467*** (0.0473)	0.468*** (0.0473)	0.507*** (0.0420)
1932	0.447*** (0.0408)	0.448*** (0.0408)	0.447*** (0.0413)	0.440*** (0.0452)	0.440*** (0.0452)	0.444*** (0.0467)
1933	0.347*** (0.0463)	0.347*** (0.0466)	0.347*** (0.0466)	0.340*** (0.0504)	0.340*** (0.0504)	0.362*** (0.0510)
1934	0.124** (0.0566)	0.124** (0.0571)	0.124** (0.0571)	0.112* (0.0587)	0.112* (0.0587)	0.0987 (0.0658)
1935	−0.246*** (0.0660)	−0.246*** (0.0667)	−0.249*** (0.0668)	−0.262*** (0.0717)	−0.261*** (0.0717)	−0.252*** (0.0726)
1936	−0.718*** (0.0679)	−0.718*** (0.0687)	−0.721*** (0.0687)	−0.730*** (0.0721)	−0.730*** (0.0721)	−0.745*** (0.0773)
1937	−1.142*** (0.0758)	−1.143*** (0.0766)	−1.142*** (0.0765)	−1.153*** (0.0770)	−1.152*** (0.0770)	−1.166*** (0.0888)
1938	−1.512*** (0.0968)	−1.513*** (0.0977)	−1.513*** (0.0977)	−1.533*** (0.0948)	−1.533*** (0.0948)	−1.530*** (0.109)
1939	−1.848*** (0.116)	−1.850*** (0.117)	−1.854*** (0.118)	−1.873*** (0.114)	−1.872*** (0.114)	−1.845*** (0.136)
1940	−2.284*** (0.108)	−2.286*** (0.109)	−2.290*** (0.110)	−2.326*** (0.109)	−2.326*** (0.109)	−2.298*** (0.126)
Father’s occupational score N/A		−0.0452*** (0.0151)	−0.0452*** (0.0151)	0.0398** (0.0157)	0.0398** (0.0156)	0.0456** (0.0180)
0–20		ref	ref	ref	ref	ref
21–40		0.0105 (0.00732)	0.0105 (0.00733)	0.0551*** (0.0171)	0.0552*** (0.0170)	0.0563** (0.0227)
41–80		0.0566*** (0.0152)	0.0567*** (0.0152)	0.104*** (0.0253)	0.104*** (0.0252)	0.102*** (0.0256)
Race: white		0.145*** (0.0260)	0.145*** (0.0260)	0.139*** (0.0284)	0.139*** (0.0284)	0.141*** (0.0283)
Place of residence: urban, non-farm		ref	ref	ref	ref	ref
Urban, farm		0.0922 (0.0637)	0.127* (0.0675)	0.101 (0.0660)	0.100 (0.0660)	0.206** (0.0848)
Rural, non-farm		−0.00645 (0.00837)	−0.00648 (0.00852)	−0.00620 (0.00918)	−0.00611 (0.00917)	−0.0134 (0.00938)
Rural, farm		0.0307* (0.0156)	0.0373** (0.0150)	0.0365** (0.0164)	0.0360** (0.0164)	0.0255 (0.0176)
Mother’s age at individual’s birth: ≤ 18		−0.0264** (0.0127)	−0.0263** (0.0127)			−0.0255 (0.0156)
19–21		−0.0101 (0.0105)	−0.0101 (0.0105)	−0.0105 (0.0128)	0.0101 (0.0180)	−0.00966 (0.0134)
22–30		ref	ref	ref	ref	ref
31–45		−0.0269*** (0.00771)	−0.0269*** (0.00770)	−0.00657 (0.00896)	0.0123 (0.00861)	−0.0287*** (0.00821)
Constant	65.66*** (0.0335)	65.57*** (0.0435)	65.56*** (0.0428)	65.53*** (0.0496)	65.51*** (0.0510)	65.53*** (0.0527)
Observations	2,419,343	2,419,343	2,419,343	2,199,294	2,199,294	1,828,832
R–squared	0.021	0.021	0.021	0.021	0.021	0.021

*Notes*: Robust standard errors in parentheses.

* p < 0.1,

** p < 0.05,

*** p < 0.01.

Models 2–5 also include state-of-birth fixed effects; not shown.

Model 5 is restricted to individuals born in states for which we have information on age restriction on cigarette purchase.

Model 6 is restricted to individuals residing in states having at least one neighboring state that had yet to implement cigarette taxation.

**Table A-2:** Brother fixed effect OLS estimates

	Model 1	Model 2
Early life exposure (state cigarette tax implemented during year of birth or year after birth)	0.184*** (0.061)	0.184*** (0.061)
Constant	65.720*** (0.459)	64.197*** (1.014)
State-of-birth FE	Y	Y
Year-of-birth FE	Y	Y
Father’s occupational score	N	-
Place/type of residence	N	-
Race	N	-
Mother’s age at individual’s birth	N	Y
Observations	249,272	249,272
Family units	121,581	121,581
Within R-squared	0.015	0.015

*Notes*: Robust standard errors in parentheses.

* p < 0.1,

** p < 0.05,

*** p < 0.01.

## Appendix B

We generated three additional datasets using publicly available linked datasets to investigate the robustness of our main results. Post-stratification weights were again used so that each analytical sample would reflect the composition of the underlying population. The first dataset was generated using Censoc data, providing links between death records from the Numident/SSDMF and the 1940 census. The results are presented in Table B-1, with the sample in Column A imposing a restriction similar to that in the main analysis, namely that members of all birth cohorts are observed to die in the same age interval (55–65 due to data coverage). Yielding a sample of 984,250 individuals, the results from both minimally and fully adjusted models produce smaller estimates than in the main analysis, as well as being statistically insignificant. It deserves to be underlined, however, that restricting the main sample to this age range yields similar results, through smaller point estimates and statistical insignificance (Column A, Table B-4). In Column B, the age restriction on the Censoc sample is relaxed, resulting in point estimates that are both larger and statistically significant. The lower point estimate compared to the main results also resembles estimates obtained on the main sample without any age restriction, thus including individuals dying at older and younger ages. Indeed, according to the fully adjusted model on the main sample, without age restrictions in Column B of Table B-4, early life exposure to state-level cigarette taxation yields a point estimate of 0.095. Consequently, while differences between the results obtained from the different samples should not be disregarded, it would appear that they are largely driven by how the analytical sample is restricted in terms of the age-at-death range.

**Table B-1:** Censoc sample OLS estimates

	(A)		(B)	
	Censoc sample		Censoc sample, no age-at-death restriction	
Variables	Minimally adjusted	Fully adjusted	Minimally adjusted	Fully adjusted
Early life exposure	0.0506 (0.0339)	0.0505 (0.0338)	0.0754*** (0.0273)	0.0723** (0.0275)
Constant	60.50*** (0.0205)	60.49*** (0.0235)	74.12*** (0.0215)	73.82*** (0.0380)
Observations	984,250	984,250	4,000,756	4,000,756
R-squared	0.010	0.010	0.318	0.318

*Notes*: Robust standard errors in parentheses.

* p < 0.1,

** p < 0.05,

*** p < 0.01.

A downside to using only the Censoc data is that information on individuals’ demographic and socioeconomic backgrounds is obtainable only from the 1940 census. Consequently, in particular for the earliest included birth cohorts, information on characteristics such as mother’s age and place of residence while in the parental household is not available. To obtain such information on individuals born before 1930, we use the Census Linkage Project’s 1930–1940 census crosswalk to link 1940 census records to 1930 census records, through the ABE exact standard algorithm. The results are displayed in Table B-2, both with the age 55–65 age-at-death restriction (Column A) and without it (Column B), with results almost identical to the baseline Censoc sample.

**Table B-2:** Censoc–ABE sample OLS estimates

	(A)		(B)	
	Censoc–ABE sample		Censoc–ABE sample, no age-at-death restriction	
Variables	Minimally adjusted	Fully adjusted	Minimally adjusted	Fully adjusted
Early life exposure	0.0611 (0.0397)	0.0611 (0.0396)	0.0855*** (0.0303)	0.0790** (0.0304)
Constant	60.48*** (0.0223)	60.50*** (0.0235)	74.39*** (0.0285)	74.18*** (0.0499)
Observations	789,869	789,869	2,922,637	2,922,637
R-squared	0.011	0.011	0.330	0.330

*Notes*: Robust standard errors in parentheses.

* p < 0.1,

** p < 0.05,

*** p < 0.01.

Lastly, we instead use 1930–1940 links published by IPUMS to attach information from the 1930 census to the cohorts born prior to 1930. The results, in Table B-3, are again nearly identical to those already presented.

**Table B-3:** Censoc–MLP sample OLS estimates

	(A)		(B)	
	Censoc–MLP sample		Censoc–MLP sample, no age-at-death restriction	
Variables	Minimally adjusted	Fully adjusted	Minimally adjusted	Fully adjusted
Early life exposure	0.0516 (0.0342)	0.0514 (0.0342)	0.0783** (0.0295)	0.0709** (0.0298)
Constant	60.48*** (0.0206)	60.47*** (0.0228)	74.19*** (0.0239)	73.91*** (0.0482)
Observations	887,206	887,206	3,446,316	3,446,316
R-squared	0.011	0.011	0.320	0.320

*Notes*: Robust standard errors in parentheses.

* p < 0.1,

** p < 0.05,

*** p < 0.01.

**Table B-4:** Main sample OLS estimates

	(A)		(B)	
	Main sample (age 55–65 age restriction)		Main sample, no age restriction	
Variables	Minimally adjusted	Fully adjusted	Minimally adjusted	Fully adjusted
Early life exposure	0.0269 (0.0208)	0.0266 (0.0208)	0.112*** (0.0389)	0.0952** (0.0398)
Constant	60.27*** (0.0161)	60.20*** (0.0215)	73.29*** (0.0595)	72.01*** (0.157)
Observations	989,392	989,392	4,392,468	4,392,468
R-squared	0.014	0.014	0.172	0.174

*Notes*: Robust standard errors in parentheses.

* p < 0.1,

** p < 0.05,

*** p < 0.01.
