# Supplementary material for: Early life exposure to cigarette smoking and adult and old-age male mortality: Evidence from linked US full-count census and mortality data
Source: Demogr Res. Author manuscript; Available in PMC 2024 Oct 11. (PMC10923319; doi:10.4054/demres.2023.49.25)
Supplement: Data info [file NIHMS1971700-supplement-Data_info.pdf]

Replication information for “Early-Life Exposure to Cigarette Smoking and Adult and Old Age Male Mortality: Evidence from Linked U.S. Full-Count Census and Mortality Data”, authored by Helgertz and Warren

The data used for the article is stored on servers maintained by the Institute for Social Research and Data Innovation (ISRDI) at the University of Minnesota. Due to their size (combined>80 GB) and containing names and Social Security Numbers, the data and do-files can be accessed directly through ISRDI. A confidentiality agreement may need to be signed, and accessing the data may require a remote connection, but is straightforward to set up.

The syntax uses several different files, with the baseline files containing the following number of observations:
1930 census: 123 million observations
1940 census: 131 million observations
1930 census – NUMIDENT file: 12 million observations
1930 census – SSDMF file: 15 million observations
1940 census – NUMIDENT file: 9 million observations
1940 census – SSDMF file: 12 million observations
Numident file: 93 million observations

The census variables are thoroughly documented at: https://usa.ipums.org/usa-action/variables/group

From the death records (SSDMF/NUMIDENT), we use both date of birth and date of death.
